# ATP-Binding Cassette Transporter G5 and G8 Polymorphisms and Several Environmental Factors with Serum Lipid Levels

**DOI:** 10.1371/journal.pone.0037972

**Published:** 2012-05-24

**Authors:** Qing Li, Rui-Xing Yin, Xian-Liang Wei, Ting-Ting Yan, Lynn Htet Htet Aung, Dong-Feng Wu, Jin-Zhen Wu, Wei-Xiong Lin, Cheng-Wu Liu, Shang-Ling Pan

**Affiliations:** 1 Department of Cardiology, Institute of Cardiovascular Diseases, The First Affiliated Hospital, Guangxi Medical University, Nanning, Guangxi, People's Republic of China; 2 Department of Anatomy, School of Premedical Sciences, Guangxi Medical University, Nanning, Guangxi, People's Republic of China; 3 Department of Molecular Biology, Medical Scientific Research Center, Nanning, Guangxi, People's Republic of China; 4 Department of Pathophysiology, School of Premedical Sciences, Guangxi Medical University, Nanning, Guangxi, People's Republic of China; INRCA, Italy

## Abstract

**Background:**

The association of ATP-binding cassette (ABC) transporter single nucleotide polymorphisms (SNPs) and serum lipid profiles is inconsistent. The present study was undertaken to detect the association of *ABCG5/G8* SNPs and several environmental factors with serum lipid levels.

**Methodology/Principal Findings:**

Genotyping of the *ABCG5* (rs4131229 and rs6720173) and *ABCG8* (rs3806471 and rs4148211) SNPs was performed in 719 unrelated subjects of Mulao nationality and 782 participants of Han nationality. There were no differences in the genotypic and allelic frequencies of four SNPs between the two ethnic groups besides the genotypic frequencies of rs4131229 SNP in Han. The levels of triglyceride (TG), apolipoprotein (Apo) A1, and ApoA1/ApoB ratio (rs4131229); low-density lipoprotein cholesterol (LDL-C) and ApoB (rs6720173); high-density lipoprotein cholesterol (HDL-C), ApoA1, ApoB, and ApoA1/ApoB ratio (rs3806471); and HDL-C, ApoA1, and ApoA1/ApoB ratio (rs4148211) in Han were different among their genotypes (*P*<0.05–0.001). The levels of LDL-C (rs6720173) and ApoA1 (rs3806471) in Mulao were also different among their genotypes (*P*<0.05 for each). The levels of TC, TG, HDL-C, ApoA1, and ApoA1/ApoB ratio (rs4131229); LDL-C and ApoB (rs6720173); HDL-C, ApoA1, and ApoA1/ApoB ratio (rs3806471); and TG, HDL-C, ApoA1, and ApoA1/ApoB ratio (rs4148211) in Han males; and ApoA1/ApoB ratio (rs4131229); LDL-C, ApoB, and ApoA1/ApoB ratio (rs3806471); HDL-C, ApoA1, and ApoA1/ApoB ratio (rs4148211) in Han females were different between the genotypes (*P*<0.05–0.001). The levels of LDL-C in Mulao females were also different between GG and GC/CC genotypes of rs6720173 (*P*<0.05). The correlation between serum lipid parameters and genotypes of four SNPs was observed in Han, especially in Han males. Serum lipid parameters were also correlated with several environmental factors.

**Conclusions:**

The associations of four *ABCG5/G8* SNPs and serum lipid levels are different between the Mulao and Han populations, or between males and females, suggesting that there may be a racial/ethnic- and/or sex-specific association between *ABCG5/G8* SNPs and some serum lipid parameters.

## Introduction

Cardiovascular disease (CVD) is the most common cause of fatality, disability and economic loss, particularly in industrialized nations. Dyslipidemia such as raised levels of total cholesterol (TC) [1], triglyceride (TG) [2], low-density lipoprotein cholesterol (LDL-C) [3], and apolipoprotein (Apo) B [4], together with decreased levels of ApoA1 [4] and high-density lipoprotein cholesterol (HDL-C) [5] is an established risk factor for CVD. Dyslipidemia is believed to be caused by various environmental and genetic factors [6], and their interactions [7]. Family history and twin studies have shown that genetic polymorphism could account for 40–70% of the interindividual variation in plasma lipid phenotypes [8], [9].

In recent years, genome-wide association (GWA) studies have identified more than 95 loci associated with serum lipid levels [10]. Common variants at these loci together can explain about 10% of variation in each lipid trait [11], [12]. Rare variants with large individual effects may also contribute to the heritability of lipid traits [12]. In addition, GWA studies also discovered a number of novel loci that influence serum lipid phenotypes [11], [13], [14]. Two of these GWA studies supported the importance of ATP-binding cassette (ABC) transporter G5 (*ABCG5*) and G8 (*ABCG8*) loci in lipid metabolism, revealing associations of serum lipid levels and CVD risk with *ABCG5* [15] and contribution of *ABCG8* to polygenic dyslipidemia [11]. The adjacent homologous genes of *ABCG5* and *ABCG8* are located in head-to-head orientation on chromosome 2p21 and the proteins are expressed exclusively in the canalicular membrane of the liver, the apical membrane of the brush border of the small intestine, and gallbladder [16], [17]. Each gene contains 13 exons encoding half-size ABC transporters with molecular weights of ∼75 kDa [18]. Rare mutations in either of the two genes that encode these transporters have been reported to cause sitosterolemia (MIM 210250; rare autosomal recessive disorder) [17], [19], characterized by high intestinal absorption of all sterols (cholesterol, plant sterols and shellfish sterols), and diminished secretion of sterols into the bile. Sitosterolemia patients have high plasma levels of cholesterol and plant sterols and develop xanthomas and premature CVD [20]. In humans, there are 48 known ABC transporters that have been linked to multidrug resistance of cancers and bacteria, cystic fibrosis, several lipid metabolism disorders, and numerous other genetic diseases [21]–[23]. Disruption of either gene in mice results in phenotypes similar to patients with sitosterolemia, such as increased plant sterol levels in plasma and tissues and decreased sterol secretion into bile [24]–[26]. Overexpression of the human genes in knock-out mice decreased sterol absorption from the diet and increased plant sterol excretion into the bile [27], [28]. Several single nucleotide polymorphisms (SNPs) in the *ABCG5* and *ABCG8* have been found to be associated with alterations of plasma or serum lipid concentrations in some studies but not in others [29]–[44]. The major reason for this inconsistency among studies may be different genetic background, sex, health status, environmental factors and their interactions to influence serum lipid levels. Thus, further study will be required to characterize the full impact of these SNPs on lipid metabolism in different racial/ethnic groups.

China is a multiethnic country with 56 ethnic groups. Han nationality is the largest ethnic group and Mulao nationality is the twenty-ninth largest minority among the 55 minority groups according to the population size of 207,352 in 2000 (the fifth national census statistics of China). Ninety percent of them live in the Luocheng Mulao Autonomous County, Guangxi Zhuang Autonomous Region, People's Republic of China. The history of this minority can be traced back to the Jin Dynasty (AD 265–420). In a previous study, Xu et al. [45] showed that the genetic relationship between Mulao nationality and other minorities in Guangxi was much closer than that between Mulao and Han or Uighur nationality. To our knowledge, however, the association of *ABCG5/G8* SNPs and serum lipid levels has not been previously reported in this population. Therefore, the aim of the present study was to explore the association of *ABCG5* (rs4131229, i7892 T>C and rs6720173, Q604E G>C) and *ABCG8* (rs3806471, 5U145 A>C and rs4148211, Y54C A>G) SNPs and several environmental factors with serum lipid profiles in the Mulao and Han populations.

## Methods

### Study populations

The present study comprised of 719 unrelated subjects of Mulao nationality who reside in Luocheng Mulao Autonomous County, Guangxi Zhuang Autonomous Region, People's Republic of China. They were randomly selected from our previous stratified randomized cluster samples. The ages of the subjects ranged from 15 to 80 years, with an average age of 51.72±14.96 years. There were 311 males (43.3%) and 408 females (56.7%). All subjects were rural agricultural workers. During the same period, a total of 782 unrelated individuals of Han nationality who reside in the same villages were also randomly selected from our previous stratified randomized cluster samples. The average age of the subjects was 51.41±15.41 years (range 15 to 80). There were 310 men (39.6%) and 472 women (60.4%). All of them were also rural agricultural workers. The subjects had no evidence of diseases related to atherosclerosis, CVD and diabetes. None of them were using lipid-lowering medication such as statins or fibrates when the blood sample was taken. The present study was approved by the Ethics Committee of the First Affiliated Hospital, Guangxi Medical University. Verbal informed consents and their thumbprints (fingerprints, to express consent) were obtained from all subjects after they received a full explanation of the study. Verbal informed consents and their parents' thumbprints of the minors/children participants involved in this study were also obtained. Written informed consents were not obtained because the educational level of the subjects was very low. The consent procedure was also approved by the Ethics Committee of the First Affiliated Hospital, Guangxi Medical University. An incentive of about ten dollars was provided to each participant in the study [7].

### Epidemiological survey

The survey was carried out using internationally standardized methods, following a common protocol [46]. Information on demographics, socioeconomic status, and lifestyle factors was collected with standardized questionnaires. The alcohol information included questions about the number of liangs (about 50 g) of rice wine, corn wine, rum, beer, or liquor consumed during the preceding 12 months. Alcohol consumption was categorized into groups of grams of alcohol per day: 0 (nondrinker), ≤25 and >25. Smoking status was categorized into groups of cigarettes per day: 0 (nonsmoker), ≤20 and >20. At the physical examination, several parameters including body height, weight, and waist circumference were measured. Sitting blood pressure was measured three times with the use of a mercury sphygmomanometer while participants were seated and had rested for at least 5 min, and the average of the three measurements was used for the level of blood pressure. Systolic blood pressure was determined by the first Korotkoff sound, and diastolic blood pressure by the fifth Korotkoff sound. Weight was measured with a portable balance scale and height with a portable steel measuring device. Body mass index (BMI) was calculated as weight in kilograms divided by the square of height in meters. Waist circumference was measured with a nonstretchable measuring tape, at the level of the smallest area of the waist, to the nearest 0.1 cm.

### Serum lipid determinations

A venous blood sample of 5 ml was drawn after fasting overnight. A part of the sample (2 ml) was collected into glass tubes and used to determine serum lipid levels. Another part of the sample (3 ml) was transferred to tubes with anticoagulate solution (4.80 g/l citric acid, 14.70 g/l glucose, and 13.20 g/l tri-sodium citrate) and used to extract deoxyribonucleic acid (DNA). Measurements of serum TC, TG, HDL-C, and LDL-C levels in the samples were performed by enzymatic methods with commercially available kits (RANDOX Laboratories Ltd., Ardmore, Diamond Road, Crumlin Co. Antrim, United Kingdom, BT29 4QY; Daiichi Pure Chemicals Co., Ltd., Tokyo, Japan). Serum ApoA1 and ApoB levels were detected by the immunoturbidimetric immunoassay using a commercial kit (RANDOX Laboratories Ltd.). All determinations were performed with an autoanalyzer (Type 7170A; Hitachi Ltd., Tokyo, Japan) in the Clinical Science Experiment Center of the First Affiliated Hospital, Guangxi Medical University.

### Genetic analyses

Total genomic DNA of the samples was isolated from peripheral blood leukocytes according to a standard phenol-chloroform method [47]–[49]. The extracted DNA was placed in long-term storage at −80°C. Genotypes of the four SNPs were determined using modified polymerase chain reaction and restriction fragment length polymorphism (PCR-RFLP). The SNPs were selected using two criteria: bioinformatics functional assessment and linkage disequilibrium (LD) structure. Computational analysis of *ABCG5/G8* SNPs (http://www.ncbi.nlm.nih.gov/SNP/buildhistory.cgi) ascribed potential functional characteristics to each variant allele. In addition, the four SNPs selected for genotyping also based on the frequency of Beijing Han population from the Human Genome Project Database. The heterozygosity values were higher than 10% for the minor allele frequency. Transform bases were used for the genotyping. The sequences of the forward and backward primers, restriction enzymes used and the size of the restriction fragments are shown in Table 1. Each reaction system of a total volume of 25 µl, comprised 100 ng (2 µl) of genomic DNA; 1.0 µl of each primer (10 µmo1/l);12.5 µl 2× *Taq* PCR MasterMix (constituent: 0.1 U *Taq* polymerase/µl, 500 µM dNTP each and PCR buffer) and nuclease-free water 8.5 µl. For the amplification, initial denaturation at 95°C for 5 min was followed by 33 cycles of denaturation at 95°C for 45 s, annealing at 59–63°C for 45 s, and extension at 72°C for 1 min, with final extension at 72°C for 10 min. After electrophoresis on a 2.0% agarose gel with 0.5 µg/ml ethidium bromide, the amplifican products were visualized under ultraviolet light. Then each restriction enzyme reaction was performed with 6 µl of amplified DNA; nuclease-free water 7.5 µl and 1 µl of 10× buffer solution; and 5 U restriction ezyme in a total volume of 15 µl digested at 37°C overnight. After restriction enzyme digestion of the amplified DNA, the digestive products were separated by electrophoresis on sepharose gel. The length of each digested DNA fragment was determined by comparing migration of a sample with that of standard DNA marker. Stained with ethidium bromide, the gel was visualized under ultraviolet light and photographed. Genotypes were scored by an experienced reader blinded to epidemiological data and serum lipid levels.

**Table 1:** The sequences of forward and backward primers, restriction enzymes for genotyping of the *ABCG5/G8* SNPs.

SNP	Primer sequence	PCR product	Restriction enzyme/site	Restriction fragment	Allele
*ABCG5* rs4131229	5′-ACTTCTGTCTGTGGCTTGCATT-3′	203 bp	*Hha* I	203	T
	5′-GAAAGAGATTGGAACCTAGAAG-3′		5′-GCG!C-3′	126+77	C
*ABCG5* rs6720173	5′-GGGCAGGTTTTCTCAATGAATTGAATTCCT**C(T)**-3′ ^X^	114 bp	*Xho* I	114	G
	5′-AACCACACCTGACACTGTCAATCTTTTCC-3′		5′-C!TCGAG-3′	83+31	C
*ABCG8* rs3806471	5′-CCATGGGGCCCACAGGTC**G(T)**GTG-3′ ^Y^	228 bp	*Ban* I	228	C
	5′-ACCATTATCTGATGTACCTTTAGCC-3′		5′-G!GYRCC-3′	209+19	A
*ABCG8* rs4148211	5′-AGGGCCTCCAGGATAGATTGTTCTCCTC-3′	128 bp	*Bgl* I	128	A
	5′-CCTTGAACCCAGGCGTGC**GC(CT)**CTACCTG-3′ ^Z^		5′-GCCNNNN!NGGC-3′	106+22	G

**X,** mismatching nucleotides (T modified C) in forward primer, *Xho* I could find the restriction site (5′-C!TCGAG-3′) in the amplicon sequence. **Y,** mismatching nucleotides (T modified G) in forward primer, *Ban* I could find the restriction site (5′-G!GYRCC-3′) in the amplicon sequence. **Z,** mismatching nucleotides (CT modified GC) in backward primer, *Bgl* I could find the restriction site (5′-GCCNNNN!NGGC-3′) in the amplicon sequence.

### DNA sequencing

Twenty-four samples (each genotype in two) detected by the PCR-RFLP were also confirmed by direct sequencing. The PCR products were purified by low melting point gel electrophoresis and phenol extraction, and then the DNA sequences were analyzed by using an ABI Prism 3100 (Applied Biosyatems) in Shanghai Sangon Biological Engineering Technology & Services Co., Ltd., People's Republic of China.

### Diagnostic criteria

The normal values of serum TC, TG, HDL-C, LDL-C, ApoA1, ApoB levels and the ratio of ApoA1 to ApoB in our Clinical Science Experiment Center were 3.10–5.17, 0.56–1.70, 1.16–1.42, 2.70–3.10 mmol/l, 1.20–1.60, 0.80–1.05 g/l, and 1.00–2.50; respectively. The individuals with TC>5.17 mmol/l and/or TG>1.70 mmol/l were defined as hyperlipidemic [47]–[49]. Hypertension was diagnosed according to the criteria of 1999 World Health Organization-International Society of Hypertension Guidelines for the management of hypertension [50], [51]. The diagnostic criteria of overweight and obesity were according to the Cooperative Meta-analysis Group of China Obesity Task Force. Normal weight, overweight and obesity were defined as a BMI<24, 24–28, and >28 kg/m^2^; respectively [52].

### Statistical analysis

Epidemiological data were recorded on a pre-designed form and managed with Excel software. The statistical analyses were done with the statistical software package SPSS 15.0 (SPSS Inc., Chicago, Illinois) or SAS 9.1 (SAS Institute, Inc., Cary, North Carolina, USA). Data are presented as mean ± standard deviation for continuous variables (serum TG levels are presented as medians and interquartile ranges) and as frequencies or percentages for categorical variables. Allele frequency was determined via direct counting, and the standard goodness-of-fit test was used to test the Hardy-Weinberg equilibrium. Difference in genotype distribution between the groups was estimated by using the chi-square test. The difference in general characteristics between two ethnic groups was tested by the Student's unpaired *t*-test. The associations of genotypes and serum lipid parameters were determined using analysis of covariance (ANCOVA). The confounding factors such as age, sex, BMI, blood pressure, alcohol consumption, and cigarette smoking were adjusted for the statistical analysis. Pair-wise linkage disequilibria (LD) among the five SNPs were estimated as correlation coefficients (*r* ^2^) using the HelixTree program (GOLDEN Helix, Bozeman, MN, USA). In order to assess the association of serum lipid levels with genotypes (rs4131229: CC = 1, CT = 2 and TT = 3; rs6720173: GG = 1, GC = 2 and CC = 3; rs3806471: CC = 1, CA = 2 and AA = 3; and rs4148211: GG = 1, GA = 2 and AA = 3) and several environment factors, multivariable linear regression analyses with stepwise modeling were also performed in the combined population of Mulao and Han, Mulao, Han, males, and females; respectively. Two-sided *P* values<0.05 were considered statistically significant.

## Results

### General and biochemical characteristics of the subjects

The general and biochemical characteristics between Mulao and Han nationalities are summarized in Table 2. The levels of body height, TG, ApoB, and the percentages of subjects who consumed alcohol were higher but the levels of BMI were lower in Mulao nationality than in Han nationality (*P*<0.05–0.001). There were no significant differences in the levels of age, weight, waist circumference, systolic blood pressure, diastolic blood pressure, pulse pressure, blood glucose, TC, LDL-C, HDL-C, ApoA1; the ratio of ApoA1 to ApoB; the percentages of subjects who smoked cigarettes; and the ratio of male to female between the two ethnic groups (*P*>0.05 for all).

**Table 2:** Comparison of general characteristics and serum lipid levels in the Mulao and Han populations.

Parameter	Han	Mulao	*t* ( 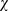 ^2^)	*P*
Number	782	719	–	–
Male/female	310/472	311/408	2.015	0.156
Age (years)	51.41±15.41	51.72±14.96	−0.388	0.698
Height (cm)	154.40±7.82	155.22±8.06	−1.972	0.049
Weight (kg)	53.39±8.91	52.87±9.35	1.110	0.267
Body mass index (kg/m^2^)	22.37±3.35	21.88±3.06	2.985	0.003
Waist circumference (cm)	75.25±7.75	75.07±8.77	0.455	0.649
Cigarette smoking [n (%)]				
Nonsmoker	580 (74.2)	542 (75.4)		
≤20 cigarettes/day	176 (22.5)	147 (20.4)	1.535	0.464
>20 cigarettes/day	26 (3.3)	30 (4.2)		
Alcohol consumption [n (%)]				
Nondrinker	622 (79.5)	550 (76.5)		
≤25 g/day	74 (9.5)	59 (8.2)	6.421	0.040
>25 g/day	86 (11.0)	110 (15.3)		
Systolic blood pressure (mmHg)	129.23±18.64	129.35±21.53	−0.105	0.916
Diastolic blood pressure (mmHg)	82.04±10.97	81.05±11.63	1.697	0.090
Pulse pressure (mmHg)	47.20±14.16	48.32±16.10	−1.418	0.516
Blood glucose (mmol/L)	6.06±1.60	5.98±1.58	1.062	0.288
Total cholesterol (mmol/L)	4.97±1.12	4.97±1.15	0.006	0.995
Triglyceride (mmol/L)	1.05 (0.89)	1.10(0.79)	−2.268	0.023
HDL-C (mmol/L)	1.73±0.53	1.74±0.46	−0.133	0.894
LDL-C (mmol/L)	2.86±0.87	2.92±0.84	−1.385	0.166
Apolipoprotein (Apo) A1 (g/L)	1.33±0.26	1.31±0.39	1.457	0.145
ApoB (g/L)	0.85±0.20	0.98±0.58	−5.829	0.000
ApoA1/ApoB	1.66±0.50	1.61±1.01	1.179	0.239

HDL-C, high-density lipoprotein cholesterol; LDL-C, low-density lipoprotein cholesterol. The value of triglyceride was presented as median (interquartile range). The difference between the two ethnic groups was determined by the Wilcoxon-Mann-Whitney test.

### Electrophoresis and genotyping

**Figure 1:**
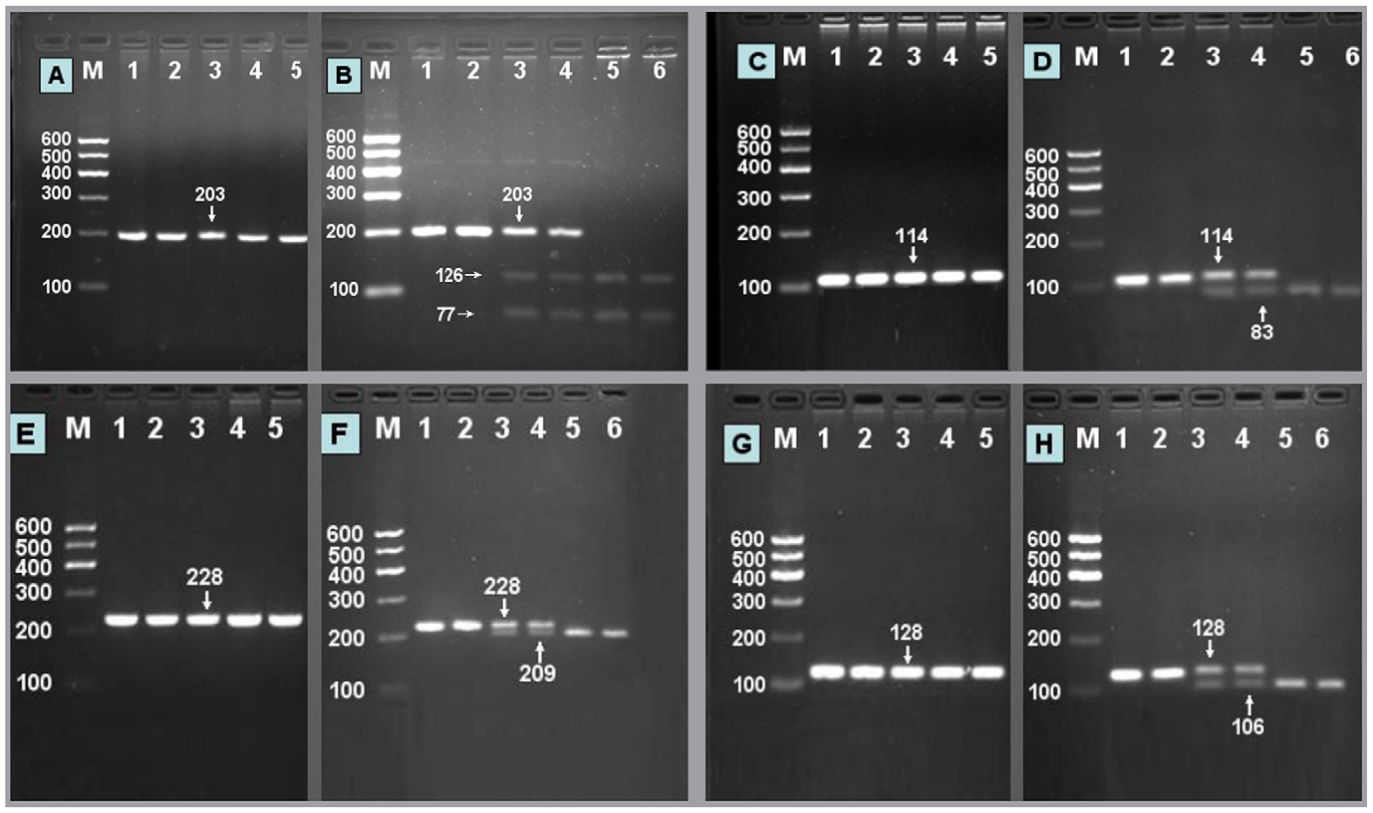
Electrophoresis of PCR products of the samples and genotyping of the *ABCG5/G8* SNPs. (A) *ABCG5* rs4131229 SNP: lane M, 100 bp marker ladder, lanes 1–5, samples, the 203 bp bands are the PCR products. (B) genotyping of the *ABCG5* rs4131229 SNP: lane M, 100 bp marker ladder; lanes 1 and 2, CC genotype (203 bp); lanes 3 and 4, CT genotype (203-, 126- and 77-bp); and lanes 5 and 6, TT genotype (126- and 77-bp). (C) *ABCG5* rs6720173 SNP: lane M, 100 bp marker ladder, lanes 1–5, samples, the 114 bp bands are the PCR products. (D) genotyping of the *ABCG5* rs6720173 SNP: lane M, 100 bp marker ladder; lanes 1 and 2, GG genotype (114 bp); lanes 3 and 4, GC genotype (114-, 83- and 31-bp); and lanes 5 and 6, CC genotype (83- and 31-bp). The 31 bp fragment was invisible in the gel owing to its fast migration speed. (E) *ABCG8* rs3806471 SNP: lane M, 100 bp marker ladder, lanes 1–5, samples, the 228 bp bands are the PCR products. (F) genotyping of the *ABCG8* rs3806471 SNP: lane M, 100 bp marker ladder; lanes 1 and 2, CC genotype (228 bp); lanes 3 and 4, CA genotype (228-, 209- and 19-bp); and lanes 5 and 6, AA genotype (209- and 19-bp). The 19 bp fragment was invisible in the gel owing to its fast migration speed. (G) *ABCG8* rs4148211 SNP: lane M, 100 bp marker ladder, lanes 1–5, samples, the 128 bp bands are the PCR products. (H) genotyping of the *ABCG8* rs4148211 SNP: lane M, 100 bp marker ladder; lanes 1 and 2, GG genotype (128 bp); lanes 3 and 4, GA genotype (128-, 106- and 22-bp); and lanes 5 and 6, AA genotype (106- and 22-bp). The 22 bp fragment was invisible in the gel owing to its fast migration speed.

After the genomic DNA of the samples was amplified by PCR and imaged by agarose gel electrophoresis, the PCR products of the SNPs could be seen in the samples (Figure 1). The genotypes of four SNPs are also shown in Figure 1.

### Results of sequencing

**Figure 2:**
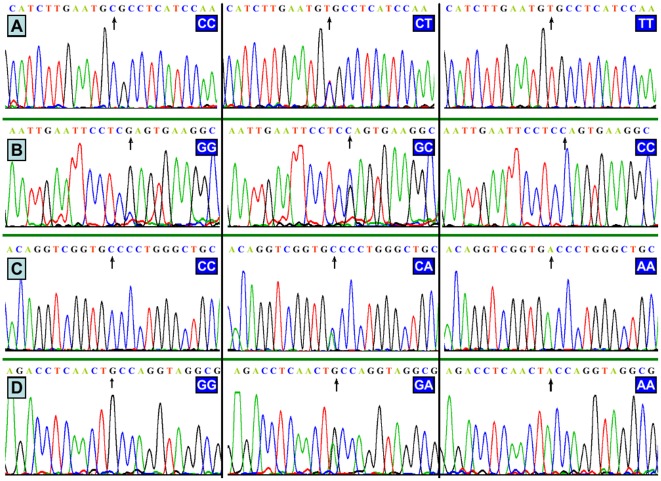
A part of the nucleotide sequences of the *ABCG5/G8* SNPs. (A) The genotypes of *ABCG5* rs4131229, (B) The genotypes of *ABCG5* rs6720173, (C) The genotypes of *ABCG8* rs3806471, and (D) The genotypes of *ABCG8* rs4148211.

All of the genotypes detected by PCR-RFLP were also confirmed by direct sequencing (Figure 2); respectively. We have deposited the raw data at Genbank's Gene Expression Omnibus (GEO) database under accession number GRP3659473.

### Genotypic and allelic frequencies

The observed genotype distribution of four SNPs was consistent with the Hardy-Weinberg equilibrium (*P*>0.05). For four SNPs, rs4131229 was in LD with rs3806471 (*r* ^2^ = 0.638, *P*<0.001) and rs4148211 (*r* ^2^ = 0.725, *P*<0.001); rs3806471 was in LD with rs4148211 (*r* ^2^ = 0.763, *P*<0.001). There was no LD between rs4131229 and rs6720173 (*r* ^2^ = 0.016, *P*>0.05), between rs6720173 and rs3806471 (*r* ^2^ = 0.013, *P*>0.05), or between rs6720173 and rs4148211 (*r* ^2^ = 0.008, *P*>0.05). Among the four examined SNPs, the frequencies of CC, CT and TT genotypes of *ABCD5* rs4131229 SNP were 70.6%, 24.0% and 5.4% in Han, and 64.8%, 30.2% and 5.0% in Mulao (*P*<0.05); respectively. There were no significant differences in the remaining genotypic and allelic frequencies between the Mulao and Han populations (Table 3). The allelic frequencies of *ABCD5* rs4131229 and the genotypic and allelic frequencies of *ABCD5* rs6720173 and *ABCD8* rs3806471 in Han but not in Mulao were different between males and females (*P*<0.05–0.01; Table 4).

**Table 3:** The genotypic and allelic frequencies of *ABCG5/G8* SNPs in the Mulao and Han populations.

SNP	Group	n	Genotype [n (%)]			Allele [n (%)]	
			AA	AB	BB	A	B
*ABCG5* rs4131229	Han	782	552 (70.6)	188(24.0)	42(5.4)	1292(82.6)	272(17.4)
	Mulao	719	466(64.8)	217(30.2)	36(5.0)	1149(79.9)	289(20.1)
	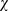 ^2^	–	7.172			3.610	
	*P*	–	0.028			0.057	
*ABCG5* rs6720173	Han	782	496(63.4)	270(34.5)	16(2.0)	1262(80.7)	302(19.3)
	Mulao	719	491(68.3)	215(29.9)	13(1.8)	1197(83.2)	241(16.8)
	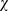 ^2^	–	3.935			3.288	
	*P*	–	0.140			0.070	
*ABCG8* rs3806471	Han	782	566(72.4)	192(24.6)	24(3.1)	1324(84.7)	240(15.3)
	Mulao	719	498(69.3)	195(27.1)	26(3.6)	1191(82.8)	247(17.2)
	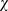 ^2^	–	1.808			1.849	
	*P*	–	0.405			0.174	
*ABCG8* rs4148211	Han	782	576(73.7)	190(24.3)	16(2.0)	1342(85.8)	222(14.2)
	Mulao	719	534(74.3)	163(22.7)	22(3.1)	1231(85.6)	207(14.4)
	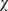 ^2^	–	1.961			0.025	
	*P*	–	0.375			0.875	

**Allele A,** rs4131229C, rs6720173G, rs3806471C, or rs4148211G. **Allele B,** rs4131229T, rs6720173C, rs3806471A, or rs4148211A. **Genotype AA,** rs4131229CC, rs6720173GG, rs3806471CC, or rs4148211GG. **Genotype AB,** rs4131229CT, rs6720173GC, rs3806471CA, or rs4148211GA. **Genotype BB,** rs4131229TT, rs6720173CC, rs3806471AA, or rs4148211AA.

**Table 4:** The genotypic and allelic frequencies of *ABCG5/G8* SNPs between males (M) and females (F) in the Mulao and Han populations.

SNP	Group	n	Genotype [n (%)]			Allele [n (%)]	
			AA	AB	BB	A	B
*ABCG5* rs4131229	Han/M	310	230(74.2)	70(22.6)	10(3.2)	530(85.5)	90(14.5)
	Han/F	472	322(68.2)	118(25.0)	32(6.8)	762(80.7)	182(19.3)
	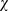 ^2^	–	5.801			5.911	
	*P*	–	0.055			0.015	
	Mulao/M	311	200(64.3)	96(30.9)	15(4.8)	496(79.7)	126(20.3)
	Mulao/F	408	266(65.2)	121(29.7)	21(5.1)	653(80.0)	163(20.0)
	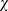 ^2^	–	0.144			0.017	
	*P*	–	0.930			0.895	
*ABCG5* rs6720173	Han/M	310	212(68.4)	94(30.3)	4(1.3)	518(83.5)	102(16.5)
	Han/F	472	284(60.2)	176(37.3)	12(2.5)	744(78.8)	200(21.2)
	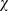 ^2^	–	6.055			5.384	
	*P*	–	0.048			0.020	
	Mulao/M	311	212(68.2)	95(30.5)	4(1.3)	519(83.4)	103(16.6)
	Mulao/F	408	279(68.4)	120(29.4)	9(2.2)	678(83.1)	138(16.9)
	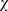 ^2^	–	0.903			0.031	
	*P*	–	0.637			0.859	
*ABCG8* rs3806471	Han/M	310	234(75.5)	74(23.9)	2(0.6)	542(87.4)	78(12.6)
	Han/F	472	332(70.3)	118(25.0)	22(4.7)	782(82.8)	162(17.2)
	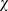 ^2^	–	10.614			6.044	
	*P*	–	0.005			0.014	
	Mulao/M	311	209(67.2)	89(28.6)	13(4.2)	507(81.5)	115(18.5)
	Mulao/F	408	289(70.8)	106(26.0)	13(3.2)	684(83.8)	132(16.2)
	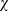 ^2^	–	1.270			1.327	
	*P*	–	0.530			0.249	
*ABCG8* rs4148211	Han/M	310	230(74.2)	78(25.2)	2(0.6)	538(86.8)	82(13.2)
	Han/F	472	346(73.3)	112(23.7)	14(3.0)	804(85.2)	140(14.8)
	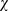 ^2^	–	5.104			0.791	
	*P*	–	0.078			0.374	
	Mulao/M	311	227(73.0)	75(24.1)	9(2.9)	592(85.0)	93(15.0)
	Mulao/F	408	307(75.2)	88(21.6)	13(3.2)	702(86.0)	114(14.0)
	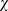 ^2^	–	0.675			0.276	
	*P*	–	0.713			0.600	

**Allele A,** rs4131229C, rs6720173G, rs3806471C, or rs4148211G. **Allele B,** rs4131229T, rs6720173C, rs3806471A, or rs4148211A. **Genotype AA,** rs4131229CC, rs6720173GG, rs3806471CC, or rs4148211GG. **Genotype AB,** rs4131229CT, rs6720173GC, rs3806471CA, or rs4148211GA. **Genotype BB,** rs4131229TT, rs6720173CC, rs3806471AA, or rs4148211AA.

### Genotypes and serum lipid levels

**Figure 3:**
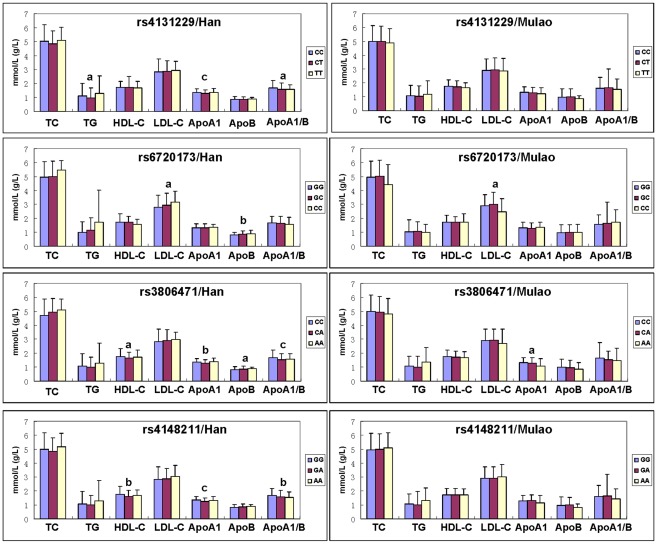
The genotypes of A*BCG5/G8* SNPs and serum lipid levels in the Mulao and Han populations. TC, total cholesterol; TG, triglyceride; HDL-C, high-density lipoprotein cholesterol; LDL-C, low-density lipoprotein cholesterol; ApoA1, apolipoprotein A1; ApoB, apolipoprotein B; ApoA1/B, the ratio of apolipoprotein A1 to apolipoprotein B. ^a^ *P*<0.05, ^b^ *P*<0.01 and ^c^ *P*<0.001.

As shown in Figure 3, the levels of TG, ApoA1, and the ratio of ApoA1 to ApoB (*ABCD5* rs4131229); LDL-C and ApoB (*ABCD5* rs6720173); HDL-C, ApoA1, ApoB, and the ratio of ApoA1 to ApoB (*ABCD8* rs3806471); and HDL-C, ApoA1, and the ratio of ApoA1 to ApoB (*ABCD8* rs4148211) in Han were different among the three genotypes (*P*<0.05–0.001). The levels of LDL-C (*ABCD5* rs6720173) and ApoA1 (*ABCD8* rs3806471) in Mulao were also different among the three genotypes (*P*<0.05 for each).

**Figure 4:**
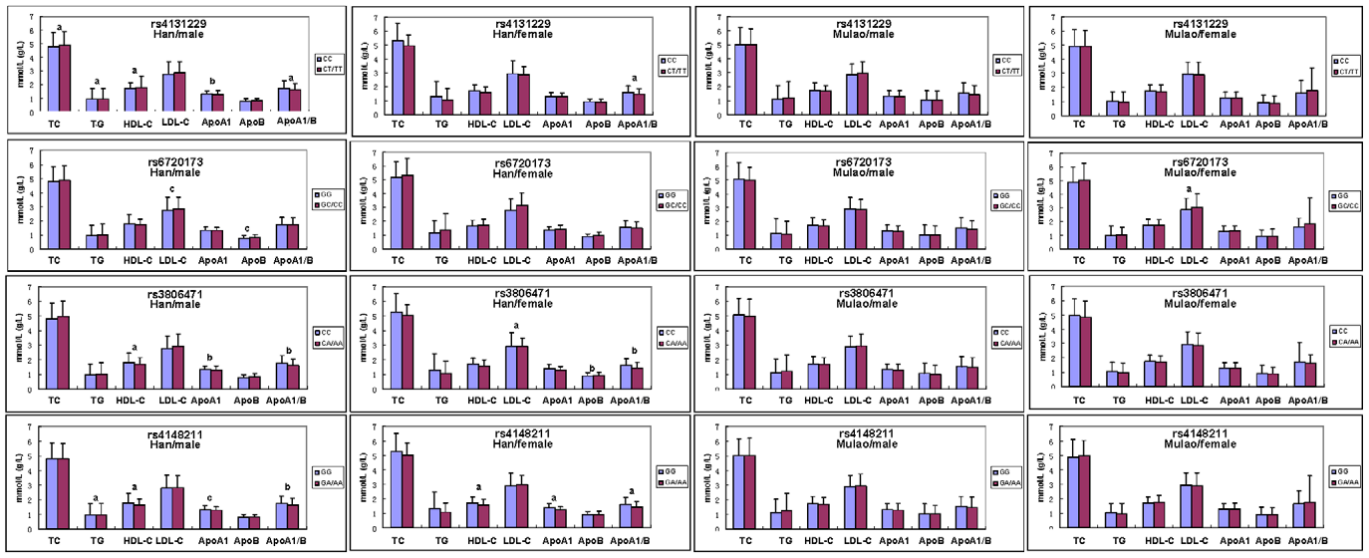
The genotypes of *ABCG5/G8* SNPs and serum lipid levels between males and females in the Mulao and Han populations. TC, total cholesterol; TG, triglyceride; HDL-C, high-density lipoprotein cholesterol; LDL-C, low-density lipoprotein cholesterol; ApoA1, apolipoprotein A1; ApoB, apolipoprotein B; ApoA1/B, the ratio of apolipoprotein A1 to apolipoprotein B. ^a^ *P*<0.05, ^b^ *P*<0.01 and ^c^ *P*<0.001.

When serum lipid levels in Han were analyzed according to sex, we found that the levels of TC, TG, HDL-C, ApoA1, and the ratio of ApoA1 to ApoB (*ABCD5* rs4131229); LDL-C and ApoB (*ABCD5* rs6720173); HDL-C, ApoA1, and the ratio of ApoA1 to ApoB (*ABCD8* rs3806471); and TG, HDL-C, ApoA1, and the ratio of ApoA1 to ApoB (*ABCD8* rs4148211) in males were different between the genotypes (*P*<0.05–0.001). The ratio of ApoA1 to ApoB (*ABCD5* rs4131229); the levels of LDL-C, ApoB, and the ratio of ApoA1 to ApoB (*ABCD8* rs3806471); HDL-C, ApoA1, and the ratio of ApoA1 to ApoB (*ABCD8* rs4148211) in females were also different between the genotypes (*P*<0.05–0.001; Figure 4).

The levels of LDL-C in Mulao females were different between the GG and GC/CC genotypes of *ABCD5* rs6720173, the subjects with GC/CC genotypes had higher serum LDL-C levels than the subjects with GG genotype (Figure 4).

### Risk factors for serum lipid parameters

The correlation between genotypes of four SNPs and serum lipid parameters is shown in Table 5. Serum lipid parameters were correlated with the genotypes in Han, especially in males. No such association was observed in Mulao.

**Table 5:** Correlation between serum lipid parameters and genotypes in the Mulao and Han populations.

Lipid	Genotype	Unstandardized coefficient	Std. error	Standardized coefficient	*t*	*P*
Han plus Mulao						
HDL-C	Genotype of rs3806471	−0.051	0.023	−0.054	−2.168	0.030
	Genotype of rs4148211	−0.050	0.025	−0.051	−2.012	0.044
ApoA1	Genotype of rs3806471	−0.037	0.016	−0.059	−2.362	0.018
	Genotype of rs4148211	−0.038	0.017	−0.058	−2.308	0.021
ApoA1/ApoB	Genotype of rs3806471	−0.097	0.037	−0.066	−2.651	0.008
Han						
HDL-C	Genotype of rs3806471	−0.075	0.036	−0.074	−2.096	0.036
	Genotype of rs4148211	−0.106	0.038	−0.099	−2.826	0.005
LDL-C	Genotype of rs6720173	0.153	0.056	0.093	2.738	0.006
ApoA1	Genotype of rs4131229	0.039	0.019	0.076	2.068	0.039
	Genotype of rs4148211	−0.053	0.018	−0.099	−2.936	0.003
ApoB	Genotype of rs6720173	0.035	0.012	0.091	2.893	0.004
ApoA1/ApoB	Genotype of rs4131229	−0.070	0.029	−0.082	−2.469	0.014
	Genotype of rs3806471	−0.106	0.031	−0.111	−3.368	0.001
	Genotype of rs4148211	−0.103	0.033	−0.102	−3.100	0.002
Han/Male						
TC	Genotype of rs4131229	−0.284	0.140	−0.107	−2.036	0.043
TG	Genotype of rs4148211	−0.880	0394	−0.116	−2.233	0.026
HDL-C	Genotype of rs4131229	−0.110	0.053	−0.112	−2.085	0.038
	Genotype of rs3806471	−0.113	0.054	−0.113	−2.100	0.037
LDL-C	Genotype of rs6720173	0.314	0.092	0.183	3.433	0.001
ApoA1	Genotype of rs4131229	−0.109	0.033	0.116	3.338	0.001
	Genotype of rs3806471	−0.94	0.033	−0.142	−2.842	0.005
	Genotype of rs4148211	−0.97	0.033	−0.149	−2.972	0.003
ApoB	Genotype of rs6720173	0.079	0.021	0.192	3.812	0.000
ApoA1/ApoB	Genotype of rs4131229	−0.127	0.055	−0.118	−2.313	0.021
	Genotype of rs3806471	−0.149	0.056	−0.136	−2.673	0.008
	Genotype of rs4148211	−0.128	0.055	−0.119	−2.313	0.021
Han/Female						
HDL-C	Genotype of rs4148211	−0.150	0.060	−0.114	−2.490	0.013
ApoB	Genotype of rs3806471	−0.044	0.017	−0.105	−2.512	0.012
ApoA1/ApoB	Genotype of rs3806471	−0.124	0.048	−0.112	−2.587	0.010

TC, total cholesterol; HDL-C, high-density lipoprotein cholesterol; LDL-C, low-density lipoprotein cholesterol; ApoA1, apolipoprotein A1; ApoB, apolipoprotein B; ApoA1/ApoB, the ratio of apolipoprotein A1 to apolipoprotein B.

Serum lipid parameters were also correlated with several environment factors such as age, gender, alcohol consumption, cigarette smoking, blood pressure, blood glucose, BMI, and waist circumference in both ethnic groups (Table 6).

**Table 6:** The environmental risk factors for serum lipid parameters in the Mulao and Han populations.

Lipid	Risk factor	Unstandardized coefficient	Std. error	Standardized coefficient	*t*	*P*
Han plus Mulao						
TC	Age	0.011	0.002	0.152	5.917	0.000
	Alcohol consumption	0.207	0.041	0.128	5.068	0.000
	Diastolic blood pressure	0.009	0.003	0.088	3.302	0.001
	Waist circumference	0.019	0.004	0.135	5.267	0.000
TG	Waist circumference	0.050	0.006	0.208	8.189	0.000
	Cigarette smoking	0.441	0.102	0.121	4.324	0.000
	Diastolic blood pressure	0.014	0.004	0.082	3.248	0.001
	Ethnic group	0.203	0.095	0.052	2.138	0.033
	Blood glucose	0.113	0.030	0.093	3.775	0.000
	Alcohol consumption	0.216	0.078	0.078	2.762	0.006
HDL-C	Waist circumference	−0.009	0.002	−0.145	−4.133	0.000
	Alcohol consumption	0.067	0.018	0.095	3.732	0.000
	Body mass index	−0.013	0.005	−0.087	−2.497	0.013
LDL-C	Age	0.010	0.001	0.183	7.352	0.000
	Body mass index	0.032	0.009	0.120	3.460	0.001
	Waist circumference	0.009	0.004	0.087	2.517	0.012
	Cigarette smoking	−0.094	0.040	−0.058	−2.322	0.020
ApoA	Alcohol consumption	0.103	0.012	0.217	8.515	0.000
	Waist circumference	−0.004	0.001	−0.088	−3.436	0.001
	Age	0.001	0.001	0.055	2.181	0.000
	Ethnic group	0.034	0.017	0.051	2.031	0.042
ApoB	Waist circumference	0.010	0.001	0.197	7.787	0.000
	Ethnic group	−0.134	0.021	−0.157	−6.357	0.000
	Blood glucose	0.025	0.007	0.094	3.760	0.000
	Cigarette smoking	0.050	0. 020	0.062	2.509	0.012
	Systolic blood pressure	0.001	0.001	0.066	2.590	0.010
ApoA1/ApoB	Waist circumference	−0.019	0.002	−0.198	−7.862	0.000
	Age	−0.005	0.001	−0.089	−3.544	0.000
Han						
TC	Waist circumference	0.019	0.005	0.131	3.767	0.000
	Age	0.009	0.003	0.126	3.443	0.001
	Alcohol consumption	0.302	0.057	0.179	5.286	0.000
	Diastolic blood pressure	0.017	0.004	0.168	4.661	0.000
	Blood glucose	0.066	0.024	0.095	2.723	0.007
TG	Waist circumference	0.075	0.013	0.254	5.661	0.000
	Cigarette smoking	0.805	0.165	0.185	4.889	0.000
	Blood glucose	0.265	0.049	0.186	5.407	0.000
	Diastolic blood pressure	0.030	0.007	0.147	4.120	0.000
	Age	−0.017	0.005	−0.113	−3.114	0.002
	Alcohol consumption	0.269	0.132	0.078	2.040	0.042
	Body mass index	−0.065	0.030	−0.096	−2.157	0.031
HDL-C	Waist circumference	−0.011	0.002	−0.155	−4.279	0.000
	Gender	0.130	0.046	0.120	2.825	0.005
	Alcohol consumption	0.111	0.034	0.138	3.317	0.001
LDL-C	Age	0.012	0.002	0.212	6.219	0.000
	Body mass index	0.026	0.012	0.101	2.222	0.027
	Waist circumference	0.013	0.005	0.115	2.471	0.014
	Cigarette smoking	−0.310	0.072	−0.187	−4.227	0.000
	Gender	−0.223	0.080	0.126	−2.802	0.005
ApoA1	Alcohol consumption	0.128	0.017	0.319	7.691	0.000
	Body mass index	−0.011	0.003	−0.136	−3.951	0.001
	Gender	0.089	0.025	0.164	3.536	0.000
	Cigarette smoking	0.072	0.023	0.141	3.154	0.002
ApoB	Waist circumference	0.005	0.001	0.207	4.741	0.000
	Blood glucose	0.022	0.004	0.168	5.102	0.000
	Alcohol consumption	0.038	0.012	0.123	3.264	0.001
	Diastolic blood pressure	0.002	0.001	0.098	2.877	0.004
	Body mass index	0.007	0.003	0.115	2.702	0.007
	Gender	−0.046	0.016	−0.111	−2.907	0.004
	Age	0.001	0.000	0.089	2.556	0.011
ApoA1/ApoB	Waist circumference	−0.010	0.003	−0.157	−3.476	0.001
	Body mass index	−0.025	0.007	−0.169	−3.814	0.000
	Cigarette smoking	0.134	0.042	0.140	3.198	0.001
	Gender	0.261	0.047	0.225	5.539	0.000
	Age	−0.004	0.001	−0.110	3.319	0.001
	Alcohol consumption	0.090	0.031	0.118	2.914	0.004
Mulao						
TC	Age	0.011	0.003	0.142	3.886	0.000
	Body mass index	0.055	0.014	0.146	3.886	0.000
	Alcohol consumption	0.148	0.057	0.095	2.595	0.010
TG	Waist circumference	0.043	0.006	0.250	6.917	0.000
	Alcohol consumption	0.221	0.073	0.105	2.915	0.004
HDL-C	Alcohol consumption	0.099	0.027	0.160	3.648	0.000
	Body mass index	−0.040	0.005	−0.265	−7.394	0.000
	Gender	0.090	0.040	0.098	2.235	0.026
LDL-C	Age	0.008	0.002	0.147	4.033	0.000
	Body mass index	0.049	0.010	0.177	4.877	0.000
ApoA1	Alcohol consumption	0.115	0.024	0.215	4.765	0.000
	Gender	0.084	0.036	0.105	2.333	0.020
Apo B	Waist circumference	0.013	0.002	0.191	5.208	0.000
	Blood glucose	0.032	0.013	0.089	2.418	0.016
ApoA1/ApoB	Age	−0.006	0.002	−0.086	−2.336	0.020
	Waist circumference	−0.019	0.004	−0.160	−4.340	0.000

TC, total cholesterol; TG, triglyceride; HDL-C, high-density lipoprotein cholesterol; LDL-C, low-density lipoprotein cholesterol; ApoA1, apolipoprotein A1; ApoB, apolipoprotein B; ApoA1/ApoB, the ratio of apolipoprotein A1 to apolipoprotein B.

## Discussion

It is well known that serum lipid levels are highly genetically determined. The heritability estimates of the interindividual variation in serum lipid phenotypes from both twin and family studies are in the range of 40–70% [8], [9], suggesting a considerable genetic contribution, and discovery of the genes that contribute to serum lipid changes may lead to a better understanding of these processes. Mulao nationality is a relatively conservative and isolated minority in China. Their engagements were family-arranged in childhood, usually with the girl being four or five years older than the boy. There was a preference for marriage to mother's brother's daughter. Engagement and marriage were marked by bride-wealth payments. Marriage ceremonies were held when the girl reached puberty. She remained with her natal family until her first child was born. Till then she was free to join the young men and women who came together for responsive singing, flirtations, and courtships at festival times. Divorce and remarriage were permitted, with little restriction. The two-generation household is the most common unit of residence. Households are under the control of the father, and divide when the sons marry, with only the youngest son remaining with the parents. Therefore, Mulao nationality is thought to share the same ethnic ancestry and to possess a homogeneous genetic background.

The genotypic and allelic frequencies of *ABCG5/G8* SNPs in different populations are inconsistent. Torres et al. [53] reported that approximately 14% of the hypercholesterolemic subjects had the *ABCG8* 52 G/C polymorphism, and 65% had the *ABCG5* 1950 C/G and G/G polymorphism. The frequency of CC homozygous genotype for *ABCG5* 1950C>G was higher in hypercholesterolemic patients than in controls (42% vs. 10%, *P*<0.001). On the other hand, no significant differences for the *ABCG8* 251A>G were observed [54]. Allele frequencies of the *ABCG8* D19H, Y54C, T400K, and A632V SNPs in patients with ischemic vascular diseases showed no significant differences compared with controls. But there was a tendency toward reduced 54YY genotype frequency among male patients with stroke. On stratification by age at disease onset, male patients with stroke under the age of 50 showed significantly reduced 54YY frequency compared with male controls (24.2% vs. 41.3%, *P* = 0.038). No such associations were found among women [55]. In the present study, we showed that the genotypic distribution of *ABCG5* rs4131229 was different between the two ethnic groups, the frequency of CT genotype was higher in Mulao than in Han (30.2% vs 24.0%, *P*<0.05). The allelic frequencies of *ABCG5* rs4131229 and the genotypic and allelic frequencies of *ABCG5* rs6720173 and *ABCG8* rs3806471 in the Han population were also different between males and females, the frequencies of minor alleles were higher in females than in males. These results indicate that the variants of *ABCG5/G8* SNPs may have a racial/ethnic-, sex- or disease-specificity.

The association of *ABCG5* SNPs and serum lipid levels is contradictory. Weggemans et al. [29] found that subjects with the EE genotype of *ABCG5* Q604E had higher serum cholesterol concentrations than carriers with the wild-type Q allele. Herron et al. [30] found that a shift from a low to a high-dietary-cholesterol diet in individuals with the *ABCG5* Q604E mutant allele was associated with a greater increase in plasma LDL-C compared to subjects who were heterozygous or who carried only the wild-type allele. Viturro et al. [31] showed that heterozygote boys had higher plasma TC, LDL-C and ApoB levels compared to homozygotes of widetype allele, but only in those within the lowest tertile of saturated fat intake. In contrast, Acalovschi et al. [32] observed the opposite effect. The 604E allele carriers had lower plasma TC levels and higher HDL-C levels compared with QQ homozygotes in 68 siblings with gallstone disease. Also Plat et al. [33] observed lower serum LDL-C levels among 604E allele carriers taking a low-erucic rapeseed oil-based margarine and shortening diet in 112 healthy Dutch volunteers. Recently, a series of GWA studies were conducted in Caucasian populations. The minor T allele of rs6756629 (R50C, C>T) in *ABCG5* was reported to be associated with lower levels of TC (*P* = 1.5×10^−11^) and LDL-C (*P* = 2.6×10^−10^) compared to the major allele [15]. However, other studies did not observe any associations between these SNPs with serum lipid parameters [34]–[39]. More recently, Garcia-Rios et al. [56] found that carriers of the minor A allele at the *ABCG5*_i11836G>A SNP displayed significantly higher HDL-C concentrations (*P* = 0.023) than G/G subjects. In addition, carriers of the minor G allele at the *ABCG5*_Gln604Glu C>G SNP had significantly lower VLDL-C (*P* = 0.011) and lower TG (*P* = 0.017) concentrations than homozygous C/C. Interestingly, a significant gene-smoking interaction was also found, in which carriers of the minor alleles at *ABCG5* (i7892A>G, i18429C>T, i11836G>A) SNPs displayed significantly lower HDL-C, higher TC and TG respectively, only in smokers. On the other hand, nonsmokers carriers of the minor alleles at *ABCG5* (i18429C>T and Gln604Glu C>G) SNPs had significantly lower TG concentrations (*P* = 0.012 and *P* = 0.035) compared with homozygous for the major allele. In the current study, we showed that the levels of TG, ApoA1, and the ratio of ApoA1 to ApoB in Han but not in Mulao were different among the CC, CT and TT genotypes of *ABCG5* rs4131229. These findings were restricted to males but not females. The levels of TC, TG, HDL-C, ApoA1, and the ratio of ApoA1 to ApoB in Han were different between CC and CT/TT genotypes only in males (*P*<0.05–0.001), the T allele carriers had lower serum levels of TC, TG, HDL-C, ApoA1 and the ratio of ApoA1 to ApoB than the T allele noncarriers. These serum lipid parameters in Han were also associated with genotypes in males but not in females. The levels of LDL-C in Mulao females and LDL-C and ApoB in Han males were different between GG and GC/CC genotypes of *ABCG5* rs6720173 SNP, the C allele carriers had higher LDL-C and ApoB than the C allele noncarriers. The levels of LDL-C and ApoB in Han were associated with genotypes in males but not in females. These findings suggest that there is a sex-specific association of *ABCG5* SNPs and some serum lipid parameters in the Han but not in the Mulao populations.

The potential associations of *ABCG8* SNPs and serum lipid levels are controversial. Several studies consistently demonstrated that subjects with the *ABCG8* 19H allele had lower serum TC [32], [36], [38], [39] and LDL-C levels [36], [38], [39] compared to the subjects without. The association of *ABCG8* D19H and LDL-C levels was recently replicated in a GWA study (*P* = 1×10^−11^) [11]. However, in another relatively big study that consisted of 2012 patients with heterozygous familial hypercholesterolemia, Koeijvoets et al. [40] did not observe any association between this SNP and lipid levels. Decreased LDL-C levels of atorvastatin treatment were not associated with the *ABCG8* D19H SNP [57]. Kajinami et al. [58] found that the *ABCG8* H19 and CYP7A1 C-204 alleles appear to interact in a dose-dependent manner on atorvastatin response. Combination analysis of these polymorphisms explained a greater percentage of LDL-C response variation than did single polymorphism analysis. The other *ABCG5/G8* SNPs (Q604E, Y54C, T400K and A632V) did not show any significant interactions with the CYP7A1 polymorphism. No association was observed between *ABCG8* T400K and total and LDL-C levels [32]–[34], [36], [38]–[41]. But the A allele carriers of *ABCG8* 1289 C>A (T400 K) with high basal plasma plant sterol concentrations demonstrated a 3.9-fold greater reduction in serum LDL-C than their low basal plasma plant sterol counterparts [59]. Regarding *ABCG8* A632V, Berge et al. [34] observed that the V allele was associated with a high plasma TC. In 380 Spanish children, Viturro et al. [31] found that the heterozygotes had higher plasma TC and ApoB levels than AA homozygotes, but only in the group with low cholesterol intake. No association of A632V and TC levels was observed in other studies [32], [33], [37]. In a recent meta-analysis comprising 3,364 subjects from 16 studies, presence of the minor 632V allele correlated with reduced LDL-C concentrations compared with homozygosity for the 632A variant (−0.11 mmol/l, *P* = 0.01). The remaining SNPs (Q604E, D19H, Y54C, and T400K) were not associated with plasma lipid levels [60]. The *ABCG8* rs4148211 SNP was associated with higher plasma TC and LDL-C concentrations in the total population. Moreover, an *ABCG5/G8* haplotype, which included the rs6544718 T allele, was associated with higher HDL-C plasma concentrations in women [61]. However, no association was observed between *ABCG8* rs4148211 and blood total and LDL-C levels in several previous studies [32], [34], [36], [38], [39]. A GWA study showed that the minor A allele of *ABCG8* rs4953023 (G>A) was associated with lower levels of LDL-C (*P* = 4×10^−8^) compared to the major allele [11]. Based on the HapMap CEU data, these SNPs are in complete LD with each other and with D19H that showed similar association result. The minor T allele of *ABCG8* rs6544713 (C>T) was associated with higher LDL-C levels (*P* = 2×10^−29^) compared to the major allele [11]. The rs6544713 SNP is not in LD with the above three SNPs (*r* ^2^<0.03), suggesting that it contributed an independent association. Common *ABCG8* SNPs were also studied in non-Caucasian populations. In 100 hypercholesterolaemic Japanese subjects, Miwa et al. [42] reported that carriers of the *ABCG8* M429V or a specific haplotype (wild-type allele of *ABCG5* Q604E, and wild-type alleles of *ABCG8* C54Y, T400K, and M429V) had higher cholesterol absorption efficiency than non-carriers. However, no difference was observed in serum lipid profiles in relation to common SNPs studied previously in Caucasian populations (*ABCG5* Q604E and *ABCG8* A632V, T400K, D19H and C54Y). This might be explained by the fact that carriers of *ABCG8* D19H and A632V SNPs are rare among Japanese compared to Caucasian populations. Interestingly, in 1046 Chinese, Chen et al. [43] observed that the heterozygote of *ABCG8* D19H had higher serum total and LDL-C levels than homozygote DD, which is opposite to the effect observed in Caucasian populations. The author speculated that this opposite effect may be due to the specific Chinese dietary pattern with high intake of plant sterols. No association with *ABCG8* C54Y and T400K regarding total and LDL-C levels was observed. *ABCG8* A632V was monomorphic in this Chinese population. For 181 hyperlipidaemic patients treated with atorvastatin (89 males), variant allele frequencies of *ABCG8* 1199A were 12.8%. For patients with the *ABCG8* C1199A variant allele, the difference in percentage reduction from baseline in TG level was increased between the CYP7A1 A-204C wild-type allele homozygotes and variant allele homozygotes after atorvastatin treatment (−28.35% vs. −19.28%, *P* = 0.001). The *ABCG8* 1199A and CYP7A1 -204A alleles appear to interact to affect lipid-lowering response to atorvastatin [62]. Recently, in 845 self-identified Puerto Ricans from Boston, Junyent et al. [38] reported that *ABCG5/G8* (i7892T>C, rs4131229; 5U145A>C, rs3806471; Y54C; T400K) SNPs were significantly associated with HDL-C concentrations. Carriers of the minor alleles at these loci and homozygotes for the T400 allele displayed lower HDL-C levels. A significant gene-smoking interaction was also found. Carriers of the minor alleles at *ABCG5/G8* (Q604E, D19H, i14222 A>G, rs6709904) SNPs displayed lower levels of HDL-C only if they were smokers. Also, for *ABCG8* T400K, smokers, but not nonsmokers, homozygous for the T allele displayed lower HDL-C levels. The result further supported a significant haplotype global effect on lowering HDL-C among smokers. The association between *ABCG5/G8* SNPs and plasma HDL-C levels possibly reconciles with an old concept: hepatobililarily excreted cholesterol mainly originates from HDL-derived cholesterol [44]. Hypercholesterolemic subjects carrying the GG genotype of the *ABCG8* 251A>G SNP also exhibited higher values of HDL-C when compared to other genotypes [54]. However, these associations were not observed in some earlier studies conducted in Caucasian populations with relatively large sample size [31], [36], [37] and also in recent GWA studies [11], [15]. Therefore, this issue needs further exploration. Recently, significant gene-gene interactions for HDL-C were found between *ABCG8* (5U145 A>C, T54C A>G, T400K C>A) SNPs and ABCA1_i48168 G>A genetic variant, in which carriers of the 5U145C and 54C alleles, and homozygotes for the T400 allele at *ABCG8* genetic variants displayed lower HDL-C concentrations than homozygotes for the 5U145A and T54 alleles, and heterozygotes for the 400K allele at *ABCG8* SNPs, only if they were also homozygous for the minor allele (A) at the aforementioned ABCA1 SNP [63]. Overall, no consistent results on effects of the *ABCG8* SNPs on serum lipid levels and cholesterol metabolic kinetics were reported so far. Failure to identify a consistent association may be due to variations in populations examined, including healthy, hypercholesterolemic, and overweight/obese subjects; modulating environmental factors such as diet or pharmaceutical treatments; or simply, a lack of power to allow for any robust conclusion to be drawn. In the present study, we showed that the levels of ApoA1 in Mulao, and HDL-C, ApoA1, ApoB, and the ratio of ApoA1 to ApoB in Han were different among the CC, CA and AA genotypes of *ABCG8* rs3806471 SNP. Subgroupanalysis showed that the levels of HDL-C and ApoA1, and the ratio of ApoA1 to ApoB in Han males, and LDL-C, ApoB, and the ratio of ApoA1 to ApoB in Han females were different among the CC, CA and AA genotypes of *ABCG8* rs3806471 SNP. The A allele carriers had lower HDL-C and ApoA1, and the ratio of ApoA1 to ApoB in Han males, and higher LDL-C, ApoB, and lower ApoA1/ApoB ratio in Han females than the A allele noncarriers. The levels of HDL-C and ApoA1, and the ratio of ApoA1 to ApoB in Han males were correlated with the genotypes, the levels of ApoB, and the ratio of ApoA1 to ApoB in Han females were associated with the genotypes. In addtion, we also showed that the levels of HDL-C, ApoA1, and the ratio of ApoA1 to ApoB in Han but not in Mulao were different among the GG, GA and AA genotypes of *ABCG8* rs4148211 SNP. On stratification by age, we showed that the levels of TG, HDL-C, ApoA1, and the ratio of ApoA1 to ApoB in Han males, and HDL-C, ApoA1, and the ratio of ApoA1 to ApoB in Han females were different among the GG, GA and AA genotypes of *ABCG8* rs4148211 SNP. The A allele carriers had lower TG, HDL-C, ApoA1, and the ratio of ApoA1 to ApoB than the A allele noncarriers. The levels of TG and ApoA1, and the ratio of ApoA1 to ApoB in Han males were correlated with the genotypes, and the levels of HDL-C in Han females were associated with the genotypes.

The extents to which serum lipid levels are affected by genetic and environmental factors remain a subject of controversy, but several environmental factors such as dietary patterns, lifestyle, obesity, physical activity, and hypertension have been shown association with serum lipid levels [6]. In the present study, we also showed that serum lipid parameters were correlated with age, sex, alcohol consumption, cigarette smoking, BMI, and blood pressure in the both ethnic groups or both sexes. These results suggest that the environmental factors and their interactions with genetic factors also play an important role in determining serum lipid levels in our populations. Although rice and corn are the staple foods in both ethnic groups, the people of Mulao nationality like to eat cold foods along with acidic and spicy dishes, so bean soy sauce and pickled vegetables are among their most popular dishes. They also like to eat animal offals which contain abundant saturated fatty acid. For nearly 50 years it has been widely accepted that high-fat diets, particularly those that contain large quantities of saturated fatty acids, raise blood cholesterol concentrations and predispose individuals to CVD. In the current study, we also found that the levels of TG and ApoB and the percentages of subjects who consumed alcohol were higher in Mulao nationality than in Han nationality. In a previous meta-analysis, 30 g of alcohol daily was associated with a plasma TG increase of 5.69 mg/dl [64]. The alcohol intake of 60 g/day increases the TG levels by about 0.19 mg/dl per 1 gram of alcohol consumed [65].

In summary, the present study shows that the associations of four *ABCG5/G8* SNPs and serum lipid levels are different between the Mulao and Han populations, or between males and females. These findings suggest that there may be a sex- and/or racial/ethnic-specific association of *ABCG5/G8* SNPs and some serum lipid parameters in our study populations.
